# Changes in the SF-8 scores among healthy non-smoking school teachers after the enforcement of a smoke-free school policy: a comparison by passive smoke status

**DOI:** 10.1186/1477-7525-8-44

**Published:** 2010-04-28

**Authors:** Kosuke Kiyohara, Yuri Itani, Takashi Kawamura, Yoshitaka Matsumoto, Yuko Takahashi

**Affiliations:** 1 Kyoto University Health Service, Yoshida-Honmachi, Sakyo-ku, Kyoto 606-8501, Japan; 2 Public Health Center, Nara City, 200-46, Nishikitsujicho, Nara 630-8325, Japan; 3 Health Administration Center, Nara Women's University, Kitauoya-Nishimachi, Nara 630-8506, Japan

## Abstract

**Background:**

The effects of the enforcement of a smoke-free workplace policy on health-related quality of life (HRQOL) among a healthy population are poorly understood. The present study was undertaken to examine the effects of the enforcement of a smoke-free school policy on HRQOL among healthy non-smoking schoolteachers with respect to their exposure to passive smoke.

**Methods:**

Two self-reported questionnaire surveys were conducted, the first before and the second after the enforcement of a total smoke-free public school policy in Nara City. A total of 1534 teachers were invited from 62 schools, and their HRQOL was assessed using six domains extracted from the Medical Outcomes Survey Short Form-8 questionnaire (SF-8): general health perception (GH), role functioning-physical (RP), vitality (VT), social functioning (SF), mental health (MH), and role functioning-emotional (RE). The participants were divided into two groups according to their exposure to environmental tobacco smoke (ETS) at baseline: participants not exposed to ETS at school (non-smokers), and participants exposed to ETS at school (passive smokers). Changes in each SF-8 score were evaluated using paired t-tests for each group, and their inter-group differences were evaluated using multiple linear regression analyses adjusted for sex, age, school type, managerial position, and attitude towards a smoke-free policy.

**Results:**

After ineligible subjects were excluded, 689 teachers were included in the analyses. The number of non-smokers and passive smokers was 447 and 242, respectively. Significant changes in SF-8 scores were observed for MH (0.9; 95% confidence interval [CI], 0.2-1.5) and RE (0.7; 95% CI, 0.0-1.3) in non-smokers, and GH (2.2; 95% CI, 1.2-3.1), VT (1.8; 95% CI, 0.9-2.7), SF (2.7; 95% CI, 1.6-3.8), MH (2.0; 95% CI, 1.0-2.9), and RE (2.0; 95% CI, 1.2-2.8) in passive smokers. In the multiple linear regression analyses, the net changes in the category scores of GH (1.8; 95% CI, 0.7-2.9), VT (1.4, 95% CI, 0.3-2.5), SF (2.5; 95% CI, 1.1-3.9), MH (1.2; 95% CI, 0.1-2.4) and RE (1.6; 95% CI, 0.5-2.7) in passive smokers significantly exceeded those in non-smokers.

**Conclusions:**

A smoke-free school policy would improve the HRQOL of healthy non-smoking teachers who are exposed to ETS.

## Background

Exposure to environmental tobacco smoke (ETS) is one of the major worldwide public health issues. Secondhand smoke is well known to definitely cause reproductive, developmental, respiratory, cardiovascular, and neoplastic diseases, as indicated in the U.S. Surgeon General's report published in 2006 [1], although its individual effects are difficult to quantify. In addition, exposure to ETS has been also reported to reduce the health-related quality of life (HRQOL) of never smokers even in the general population [2] as well as of patients with asthma [3] or chronic obstructive pulmonary diseases (COPD) [4].

One possible solution for the elimination of health hazards from ETS is to make public places smoke-free. Previous studies suggested that smoke-free workplace policies could contribute to the reduction in respiratory symptoms of workers [5,6] and heart disease morbidity/mortality [7,8]. In addition, one study also suggested that disease-specific quality of life among non-smoking asthmatic bar workers would significantly improve after the implementation of smoke-free legislation [9].

However, the effects of smoke-free legislation on HRQOL of the healthy population are still unknown. Odor annoyance and ocular/nasal irritation are well-known acute symptoms of secondhand smoke [10,11], and some acute respiratory symptoms, including coughing, wheezing, chest tightness, and breathing difficulty, might occur among healthy persons exposed to ETS [12-15]. As the U.S. Surgeon General's report mentioned, these respiratory and sensory symptoms may potentially deteriorate HRQOL [1]. Therefore, eliminating or reducing secondhand smoke would contribute to the improvement of HRQOL even for healthy persons.

The Health Promotion Law of Japan, which came into force in 2002, put the managers of facilities of a public nature, including restaurants, cafes, public transportation, schools, and offices, under an obligation to control secondhand smoke. In accordance with this legislation, the Nara City government implemented a smoke-free school policy in all public schools in April 2007. Taking this opportunity, the researchers examined how the HRQOL of subjectively healthy schoolteachers changed. The goal of the present study was to investigate the effects of the smoke-free school policy on HRQOL among healthy non-smoking schoolteachers with respect to their exposure to passive smoke.

## Methods

### Survey and participants

Two self-reported questionnaire surveys were conducted in January 2007 and September 2007, the first three months before and the second five months after the enforcement of the total smoke-free public school policy in Nara City, respectively. The questionnaire forms were sent to 1748 teachers affiliated with 70 public elementary, junior high, and senior high schools in Nara City for each survey. Since eight out of 70 schools had already adopted the smoke-free school policy of their own accord before the first survey, the 214 teachers assigned to these schools were excluded, and the remaining 1534 were enrolled in the study. Among the latter group, participants who answered both the baseline and follow-up questionnaires, had no missing values in the required questionnaire items, did not smoke at baseline, and did not have definite/suspected diseases at baseline, were eligible for the following analyses.

### Data collection

HRQOL was assessed by the Medical Outcomes Survey Short Form-8 questionnaire (SF-8) [16]. SF-8 consists of eight items, each representing one health profile dimension: general health perception (GH), physical functioning (PF), role functioning-physical (RP), bodily pain (BP), vitality (VT), social functioning (SF), mental health (MH), and role functioning-emotional (RE). Each item of the SF-8 is assessed using a 5- or 6-point Likert scale, and is standardized according to the scoring system, in which 50 points represents the national standard value for health and functioning. The Japanese version of the SF-8 meets the standard criteria for content and for construct and criterion validity, based on the national survey covering 1,000 Japanese general citizens in 2002 [16]. We chose six out of the eight items of SF-8: GH, RP, VT, SF, MH, and RE for the analyses. In addition to HRQOL, sex, age, school type, managerial position, current smoking status, experience of secondhand smoke at school during the past month, and attitude towards the smoke-free school policy were also examined in the self-report questionnaire. Attitude towards the smoke-free school policy was examined using a 5-point Likert scale (very positive, rather positive, equivocal, rather negative, and very negative).

### Statistical methods

The participants were divided into two groups according to their experience of secondhand smoke at baseline: participants not exposed to ETS (non-smokers) and participants exposed to ETS (passive smokers).

Differences in the baseline characteristics between the groups were evaluated using chi-square test, and those in the baseline scores for the SF-8 between the groups were evaluated using Student's t-test. Changes in each score between before and after the enforcement of the smoke-free policy were evaluated using paired t-test in both groups. The level of significance was set at 5%. In addition, the differences of the net changes in each category score between the groups were evaluated using multiple linear regression analysis to calculate partial regression coefficients and their 95% confidence intervals (CIs), adjusted for sex, age, school type, managerial position, and attitude towards the smoke-free school policy. All analyses were conducted with the SPSS v.15.0 J for Windows statistical software (SPSS Inc., Chicago, IL).

### Ethics

Answering the questionnaires was voluntary, and all the participants were identified by research-specific numbers after removing personal identifiers. This study protocol was approved by the ethics committee of Nara Women's University.

## Results

### Baseline characteristics of the participants

**Figure 1:**
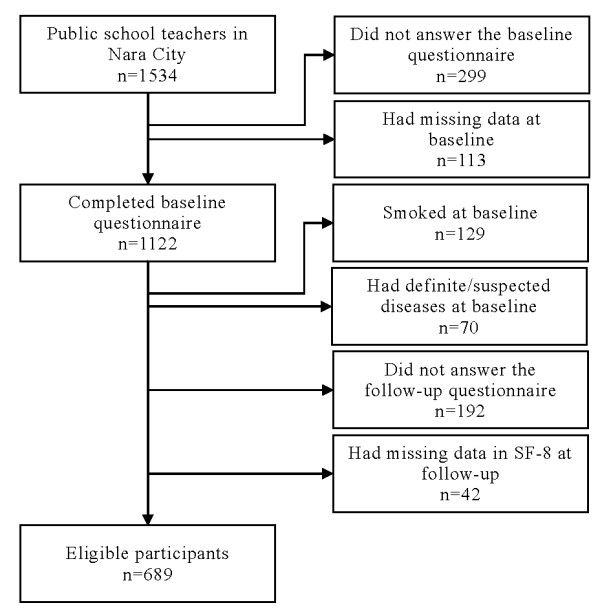
**Flowchart of the study participants**.

Figure 1 shows the flowchart of the participants included in the present study. Out of 1534 enrollees, 1122 completed the baseline questionnaire without data missing. Excluding teachers who smoked at baseline, had definite/suspected diseases at baseline, did not answer the follow-up questionnaire, and had missing data in the follow-up survey, the remaining 689 were eligible for the analyses. Compared with the eligible participants (n = 689), teachers who did not answer the follow-up questionnaire or had missing data in the SF-8 at follow-up (n = 234) were somewhat more likely to be male (106 of 234 [45%] vs 257 of 689 [37%]; p = 0.030) and had a less positive attitude towards the smoke-free school policy (173 of 234 [74%] vs 555 of 689 [81%]; p = 0.032).

After the enforcement of the smoke-free policy, 16 (14%) of the 111 smoking teachers completing the follow-up survey had quit smoking successfully.

Table 1 shows the baseline characteristics of the participants. The number of participants of non-smokers and passive smokers was 447 and 242, respectively. Passive smokers were somewhat younger (p = 0.036) and more likely to belong to junior and senior high schools (p = 0.001) compared with non-smokers. Only a few senior high school teachers (31 in number) were available because of the uniqueness of the municipal high school in Nara City.

**Table 1:** Baseline characteristics of the participants

	Total		Non-smokers*		Passive smokers**		P-value
				
	n	(%)	n	(%)	n	(%)	
Age							
<50 years old	367	(53%)	225	(50%)	142	(59%)	0.036
≥50 years old	322	(47%)	222	(50%)	100	(41%)	
							
Sex							
Male	257	(37%)	159	(36%)	98	(40%)	0.137
Female	432	(63%)	288	(64%)	144	(60%)	
							
Managerial position							
General teacher	572	(83%)	373	(83%)	199	(82%)	0.269
Principal or vice-principal	60	(9%)	42	(9%)	18	(7%)	
School nurse or dietitian	57	(8%)	32	(7%)	25	(10%)	
							
School type							
Elementary school	437	(63%)	300	(67%)	137	(57%)	0.001
Junior high school	221	(32%)	135	(30%)	86	(36%)	
High school	31	(4%)	12	(3%)	19	(8%)	
							
Attitude towards smoke-free schools							
Positive	555	(81%)	357	(80%)	198	(82%)	0.537
Not positive	134	(19%)	90	(20%)	44	(18%)	

**Total**	**689**		**447**		**242**		

*Non-smokers: Participants who were not exposed to environmental tobacco smoke at baseline

**Passive smokers: Participants who were exposed to environmental tobacco smoke at baseline

### Change in HRQOL before and after the enforcement of the smoke-free school policy

Table 2 shows the SF-8 scores at baseline and at follow-up for each group. The category scores of passive smokers at baseline were lower than those of non-smokers for GH (1.4, p = 0.013), SF (2.3, p = 0.001), MH (1.4, p = 0.011), and RE (1.6, p = 0.004). Significant increases were observed after the enforcement of the smoke-free school policy in the scores for MH (0.9; 95% CI, 0.2-1.5) and RE (0.7; 95% CI, 0.0-1.3) in non-smokers, and GH (2.2; 95% CI, 1.2-3.1), VT (1.8; 95% CI, 0.9-2.7), SF (2.7; 95% CI, 1.6-3.8), MH (2.0; 95% CI, 1.0-2.9), and RE (2.0; 95% CI, 1.2-2.8) in passive smokers.

**Table 2:** SF-8 scores before and after the enforcement of the smoke-free school policy

Group	Domain of SF-8*	Score		
			
		Baseline	Follow-up	P-value
				
		Mean ± SD	Mean ± SD	
Non-smokers	GH	48.3 ± 6.7	48.6 ± 6.7	0.304
	RP	46.8 ± 6.5	47.2 ± 6.9	0.214
	VT	47.7 ± 6.3	48.1 ± 5.9	0.256
	SF	45.8 ± 8.2	46.1 ± 7.8	0.501
	MH	46.9 ± 6.6	47.7 ± 6.5	0.013
	RE	47.1 ± 6.9	47.8 ± 6.1	0.040

Passive smokers	GH	46.9 ± 7.2	49.0 ± 7.0	<0.001
	RP	46.7 ± 6.5	47.3 ± 7.3	0.201
	VT	47.2 ± 6.8	49.0 ± 6.9	<0.001
	SF	43.6 ± 8.4	46.2 ± 8.4	<0.001
	MH	45.5 ± 7.2	47.4 ± 7.2	<0.001
	RE	45.5 ± 7.3	47.5 ± 6.9	<0.001

*GH: General health, RP: Role-physical, VT: Vitality SF: Social functioning, MH: Mental health, RE: Role-emotional

Table 3 shows the differences of the net changes in the category scores between non-smokers and passive smokers, and the regression coefficients generated by the linear regression analyses. The results of the univariable and multivariable analyses were quite similar. All of the category scores, but for RP among passive smokers, increased significantly more than those among non-smokers.

**Table 3:** Differences of the net changes in SF-8 scores between non-smokers and passive smokers

Domain of SF-8*	Net changes in SF-8 scores before and after enforcement of the smoke-free school policy		Differences of the net changes in the SF-8 scores between non-smokers and passive smokers	
		
			Univariable analysis	Multivariable analysis**
				
	Non-smokers	Passive smokers	Regression coefficient (95% CI)	Regression coefficient (95% CI)
GH	0.3	2.2	1.8 (0.7 - 3.0)	1.8 (0.7 - 2.9)
RP	0.4	0.6	0.2 (-0.9 - 1.3)	0.2 (-1.0 - 1.3)
VT	0.3	1.8	1.5 (0.4 - 2.5)	1.4 (0.3 - 2.5)
SF	0.3	2.7	2.4 (1.0 - 3.8)	2.5 (1.1 - 3.9)
MH	0.9	2.0	1.1 (0.0 - 2.2)	1.2 (0.1 - 2.4)
RE	0.7	2.0	1.3 (0.2 - 2.4)	1.6 (0.5 - 2.7)

*GH: General health, RP: Role-physical, VT: Vitality SF: Social functioning, MH: Mental health, RE: Role-emotional

** Adjusted for sex, age, school type, managerial position, and attitude towards smoke-free school policy

## Discussion

The smoke-free school policy was originally introduced to protect pupils from exposure to ETS [17]. It was also expected to encourage smoking teachers to quit or reduce their smoking [18] and to prevent pupils from starting smoking [19-21]. Our results implied that a smoke-free school policy would also contribute to improving the HRQOL of non-smoking teachers who are exposed to ETS at school. Although our follow-up study design allowed us to assess the causal relationship between the smoke-free school policy and the changes in HRQOL, this simple before-and-after comparison could not indicate when HRQOL had changed. Further time-series studies are needed to clarify this.

The baseline SF-8 scores of teachers who were regularly exposed to ETS in workplaces were lower than those of non-smokers and also lower than the Japanese National Norms [16], even though the study participants were limited to subjectively healthy persons. This finding is consistent with the previous study [2]. Referring to the studies using SF-8 reporting that patients with Japanese cedar pollinosis had a lower mental component score by 1.7 on the SF-8 than the Japanese National Norm [22], and that university students having any allergic disorders showed lower domain scores by 2.3 on the SF-8 than those having no allergy [23], the differences in the SF-8 scores between non-smokers and passive smokers at baseline were considered to be clinically relevant.

Our follow-up survey results suggest that the elimination of ETS by the enforcement of the smoke-free school policy would improve all categories of SF-8 except for RP among passive smokers, reaching identical levels to those of the non-smokers at follow-up. To our knowledge, the present study is the first follow-up survey to evaluate the effects of a social healthcare intervention using SF-8. Therefore, it is difficult to compare its efficacy with those of other social interventions.

We assessed the HRQOL of the participants using SF-8, the scores of which can be directly compared with the scores obtained from the Medical Outcomes Survey 36-item short form health survey (SF-36) [24,25], a widely-accepted scale for measuring comprehensive quality of life. A decline in the scores for SF-36 would increase the risk of death and of hospitalization [26], and the scores also predict total healthcare costs [27]. Since SF-8 is a shortened version of SF-36, its accuracy might be inferior to that of SF-36. However, the correlation coefficient of each 8-category scale score between SF-8 and SF-36 was substantially high (Spearman r = 0.56 - 0.87) [16], and it was deemed to be a suitable surrogate for evaluating HRQOL. The primary advantage of SF-8 is its simplicity, and as such, it is better suited for mass screening.

This study had some limitations in its design. First, self-reported secondhand smoke was not verified for the measure of ETS exposure in schools. Since the questionnaire survey for ETS exposure and active smoking were reported to be vulnerable to misclassification [28,29], biochemical measures, such as expiratory gas carbon monoxide and urine or blood cotinine, would be desirable. However, these methods are time-consuming and costly and cannot identify the source of secondhand smoke. The large number of the participants and the long time between the policy enforcement and the surveys should have minimized the temporary fluctuations in the answers. Second, we did not consider exposure to ETS at home or in other private places. Bridevaux et al. [2] reported that exposure to ETS at home strongly affects HRQOL. Additionally, several studies pointed out the significant relationship between one's physical activity level and HRQOL [30-34]. These factors might have confounded the results. Third, findings among teachers cannot be well generalized. The proportion of smokers at baseline (male, 29%; female, 1%) was substantially lower than that of the general population in Japan (male, 40%; female, 10%) [35]. This is probably because schoolteachers are highly educated and are expected to behave as role models for pupils. Fourth, since the baseline survey was carried out in mid-winter and the follow-up survey in early autumn, the shift in seasons might have affected HRQOL. Actually, even among teachers who were not exposed to ETS, some domain scores of the SF-8 significantly improved, though they should not be influenced by the enforcement of the smoke-free school policy. The changes in the scores might partly be seasonal effects. However, we primarily focused on the comparison between non-smokers and passive smokers, and their inter-group comparability was preserved. Fifth, we excluded two domains of the SF-8, PF and BP, from the questionnaire form. According to the SF-8 manual for Japanese, people suffering any physical disorder showed significantly lower category scores particularly in the physical-related domain, such as BP, RP, and PF, than did healthy people [16]. Since the study participants were subjectively healthy teachers, physical-related domains would have little relation to the short-term effects of smoke-free school policy. Therefore, we excluded PF and BP from the questionnaire and included only RP to check its independency. As expected, no significant changes in RP score were seen in either non-smokers or passive smokers. However, our arbitrary alternation of the standardized instrument is a methodological violation, and it would preclude a thorough interpretation of the results. As the previous study suggested a relationship between those physical-related domains and exposure to ETS among nonsmoking women [2], these domains should have been examined as well.

## Conclusions

Exposure to ETS in schools lowers HRQOL among non-smoking teachers, and the enforcement of a smoke-free school policy would improve their HRQOL. Our findings should encourage policy makers to push ahead with restricting smoking in schools.

## List of abbreviations

ETS: environmental tobacco smoke; HRQOL: health-related quality of life; COPD: chronic obstructive pulmonary disease; SF-8: Medical Outcomes Survey Short Form-8 questionnaire; GH: general health perception; PF: physical functioning; RP: role functioning physical; BP: bodily pain; VT: vitality; SF: social functioning; MH: mental health; RE: role functioning emotional; CI: confidence interval; SF-36: Medical Outcomes Survey 36-item short form health survey.

## Competing interests

The authors declare that they have no competing interests.

## Authors' contributions

KK designed the questionnaire, analyzed the data, and drafted the manuscript. YI designed the questionnaire, performed the survey, and collected and input the data. TK designed the statistical analyses and drafted the manuscript. YM designed the questionnaire and performed the survey. YT supervised the whole survey. All authors read and approved the final manuscript.
